# The Histopathology of Leg Ulcers

**DOI:** 10.3390/dermatopathology11010007

**Published:** 2024-01-29

**Authors:** Amun Georg Hofmann, Julia Deinsberger, André Oszwald, Benedikt Weber

**Affiliations:** 1 Department of Dermatology, Medical University of Vienna, Währinger Gürtel 18-20, 1090 Vienna, Austriajulia.deinsberger@meduniwien.ac.at (J.D.); 2 Department of Pathology, Medical University of Vienna, Währinger Gürtel 18-20, 1090 Vienna, Austria; andre.oszwald@meduniwien.ac.at

**Keywords:** leg ulcer, ulceration, wounds, histopathology

## Abstract

Ulcerations of the lower extremities are a frequently encountered problem in clinical practice and are of significant interest in public health due to the high prevalence of underlying pathologies, including chronic venous disease, diabetes and peripheral arterial occlusive disease. However, leg ulcers can also present as signs and symptoms of various rare diseases and even as an adverse reaction to drugs. In such cases, correct diagnosis ultimately relies on histopathological examination. Apart from the macroscopic presentation, patient history and anatomic location, which are sometimes indicative, most ulcers have very distinct histopathological features. These features are found in different layers of the skin or even associated vessels. In this narrative review, we discuss and highlight the histopathological differences of several types of leg ulcers that can contribute to efficient and accurate diagnosis.

## 1. Introduction

The term leg ulcer, referring to chronic wounds on the lower extremities, does not constitute a diagnosis itself but reflects a symptom or long-term consequence of underlying diseases [1]. Precise epidemiological data are scarce, but the reported prevalence ranges between 0.18% and 2% in the European population and may affect between 3.6% and 5% of patients aged 65 years and older globally [2,3,4,5].

The most frequent etiologies are venous insufficiency (reported share among leg ulcers: 43–85%), arterial insufficiency (10–20%), diabetes (10–15%) and a combination of these (10–15%) [1,2,6,7,8,9]. However, there are at least 180 different reported causes of leg ulcerations. Among them are other vascular causes, vasculitides, other immunological and infectious diseases, malignant tumors and hematological disorders [2].

Diagnosis is often based on clinical and/or instrument-based data, but it can be challenging when the underlying etiology is unrelated to venous or arterial pathologies. The frequency of uncommon etiologies in leg ulcers is scientifically debated, ranging from 1 to 30% [10,11,12,13].

Leg ulcers that do not respond to standard wound therapy and exhibit atypical macroscopic features or clinical manifestations should be subject to a wound biopsy and histopathological examination [12,14,15,16]. The histopathological hallmark of ulcerations is the loss of the epidermis and of at least a part of the dermis, which can also affect subcutaneous tissue. In the course of the ulcerative process, the normal pattern of collagen bundles in the dermis is destroyed, resulting in scar tissue development [17]. Additional patterns and features, such as an immune cell infiltrates or specific vascular changes, can guide the diagnosis based on the histopathological picture [18].

This review comprehensively outlines the histopathological findings observed in various types of chronic leg ulcerations, aiding in the differential diagnosis of this significant cutaneous disorder.

## 2. Venous Leg Ulcers

Chronic venous disease (CVD) is the most frequent cause of leg ulcers, underlying 43–85% of all cases [6,7,17,19]. The underlying pathophysiology of CVD is a mismatch of venous pressure and unidirectional blood flow. Dilation of the vessel wall and consequent valvular incompetence lead to reflux, which promotes venous hypertension and thus establishes a vicious circle [20,21]. CVD is commonly classified by clinical manifestation according to the CEAP system, comprising six classes (C1–C6). C6 is the most severe class and is characterized by the presence of active venous leg ulcers (VLUs) [22], most frequently affecting the medial and anterior malleolus and the pretibial area [6]. Due to the frequency and characteristic macroscopic appearance of VLU, their histopathological examination is an exception. Guidelines suggest performing a biopsy only in case of atypical features or unresponsiveness to 4 to 6 weeks of wound and compression therapy [14,15,23].

**Figure 1:**
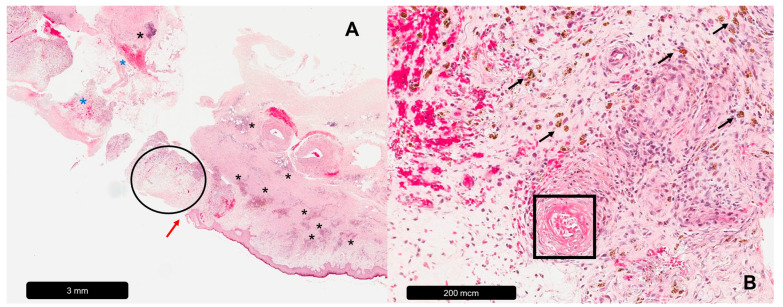
Leg ulcer caused by chronic venous disease. (**A**) Overview, red arrow = beginning of ulceration, black asterisk = inflammatory infiltrates, blue asterisk = erythrocyte extravasate, black circle = diffuse edema. (**B**) Magnification, black arrows = hemosiderophages, black rectangle = fibrin cuff.

The inflammatory infiltrates of VLU samples comprise macrophages, mast cells and lymphocytes. Interestingly, biopsies of non-ulcerated skin from CVD patients (without active ulcerations) also show increased dermal leukocyte infiltration in all stages of the disease, mostly macrophages and T-lymphocytes, suggesting perpetuated chronic skin inflammation in CVD [24,25]. The wound bed often contains necrotic tissue and/or insufficient granulation tissue [26]. The transition from a normal epidermis to an ulcer can be abrupt in VLUs, resulting in a so-called “step sign” [17]. Increased expression of the endothelial adhesion molecules ICAM-1, VCAM-1 and E-selectin has been shown at the border of the ulcer [27]. The remaining epidermis characteristically shows spongiosis, hyperkeratosis and acanthosis, and frequently observed dermal changes include diffuse edema, granulation tissue, fibrin, hemosiderophages, collagen bundle degeneration and fibrosis [17,28,29,30,31]. The appearance of pericapillary fibrin cuffs that incorporate laminin, fibronectin, tenascin, collagen and trapped leukocytes is highly characteristic of VLUs [29,32,33,34,35]. Microcirculatory dysfunction is reflected by dilated capillaries and a reduced capillary density, which has been shown to be negatively associated with VLU healing tendencies [27,35]. In the skin surrounding VLUs, lymphatic vessel density is increased. However, the vessels are more often collapsed, and the remaining patent vessels more often show open inter-endothelial junctions compared with healthy controls [36]. Impaired lymph flow may present as lymphangectasia and result in phlebolymphedema [17] (see Table 1, Figure 1).

### 2.1. Mixed Venous-Arterial Leg Ulcers

Leg ulcers with a mixed etiology are usually caused by a combination of venous insufficiency and other venous or arterial pathologies. Accordingly, most of these ulcers present with macro- and microscopical features of VLUs and additional characteristics of arterial ulcers, e.g., ischemic necrosis [6,17].

### 2.2. Livedoid Vasculopathy

**Figure 2:**
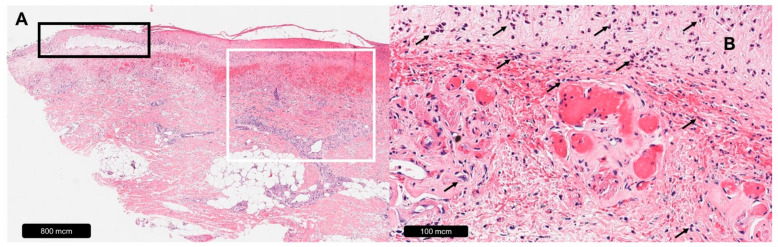
Ulcer caused by livedoid vasculopathy. (**A**) Overview, black rectangle = epidermal atrophy, white rectangle = diffuse leukocyte infiltration. (**B**) Magnification, black arrows = leukocytes.

Livedoid vasculopathy, formerly known as *atrophie blanche* due to characteristic porcelain-white scars [37], is a cutaneous disorder characterized by bilateral leg ulceration [38,39]. Its pathogenesis involves the focal non-inflammatory thrombosis of dermal veins and venulae in the subpapillary vascular plexus [39,40] that can usually be observed in histopathological examinations [41,42]. Other features include epidermal spongiosis or atrophy in the margin area, vessel wall thickening with endothelial proliferation, fibrinoid degeneration, fibrin deposits and fibrin thrombi in the lumen [38,39,43,44,45,46]. Subintimal hyalinization and the absence of perivascular inflammation are characteristic, even though secondary inflammatory changes may be observed in later stages [38,42,44,47] (see Table 2, Figure 2).

## 3. Arterial Leg Ulcers

### 3.1. Occlusive-Ischemic

Arterial leg ulcers develop due to an oxygen deficit in the tissue and are most commonly associated with peripheral artery disease (PAD) [1]. PAD is mainly caused by atherosclerotic narrowing of the arteries. Subsequent hemodynamic changes and macro- and microvascular adaptions, as well as tissue remodeling processes, impair blood supply, leading to end-organ ischemia [48]. The two most commonly used systems to classify the disease, Rutherford and Fontaine, are both based on clinical criteria. The most severe stage in both systems is based on tissue loss, as seen in ulcerations and gangrene [49]. PAD ulcers can primarily be found on the toes and plantar, presenting as demarcated lesions [50]. The wound bed tends to be dry, and necrotic areas might be macroscopically visible [30,51]. Histologically, they are highly associated with epidermal thinning and necrosis. In the dermis, thrombosed vessels and dermal sclerosis with potentially hyalinized areas can be observed [17] (see Table 3).

### 3.2. Ischemic Subcutaneous Arteriolosclerosis

Ischemic subcutaneous arteriolosclerosis describes several entities that share arteriolar calcification as one of their histopathological hallmarks. It includes proximal non-uremic calciphylaxis with normal renal and parathyroid function and proximal and distal calciphylaxis in patients with end-stage renal insufficiency, as well as the arteriolosclerotic ulcer of Martorell [1,52,53].

### 3.3. Arteriolosclerotic Ulcer of Martorell

**Figure 3:**
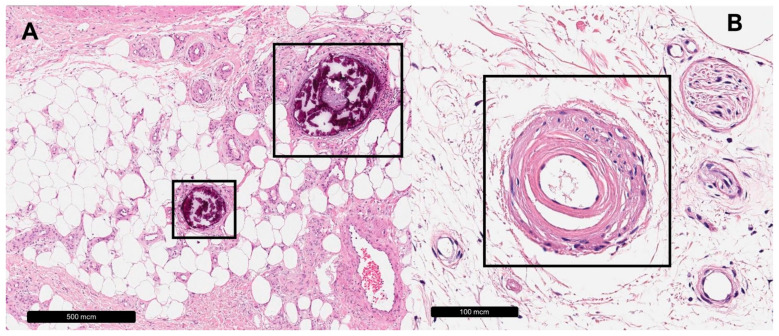
Arteriolosclerotic ulcer of Martorell. (**A**) Black rectangles = calcification. (**B**) Black rectangle = subintimal hyalinization.

The arteriolosclerotic ulcer of Martorell is typically located on the lateral side of the lower legs, characterized by disproportional pain, and it is strongly associated with arterial hypertension [54,55]. The most prominent histopathological features include media hypertrophy and progressive hyalinotic alteration of the arteriolar wall, leading to an increased wall–lumen ratio and subsequently resulting in luminal stenosis [52,56]. Additionally, arteriolar calcification can be seen in most cases [52,57,58]. Further described histopathological features include acanthosis, intimal hyperplasia, sub-endothelial hyalinosis, luminal thrombosis and necrosis [52,57,58,59,60,61,62]. The frequently observed periarteriolitis may occur as a non-specific cellular response to the surrounding necrosis [57,63] (see Table 4). Venous vessels are often severely damaged due to the inflammatory process and may develop thrombotic occlusions [64,65,66]. However, the specificity of these features is highly debated to be insufficient [57,67]. For example, it has been shown that cutaneous arteriolosclerosis is independently associated with age [68] (see Figure 3).

### 3.4. Calciphylaxis

**Figure 4:**
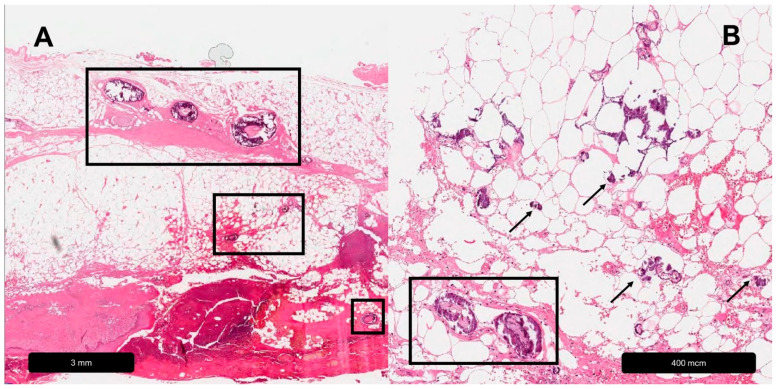
Calciphylaxis. (**A**) Overview, black rectangles = calcifications in different layers of the skin. (**B**) Magnification, black rectangle = vessel-associated calcifications, black arrows = extra-arterial calcium deposits.

Calciphylaxis is a calcifying vasculopathy of the small- and medium-sized blood vessels of the dermis and subcutis, which in turn leads to ischemia and necrosis of the affected skin areas [69]. Calciphylaxis is most frequently associated with chronic kidney failure and the related disturbance of calcium phosphate metabolism. However, there are also non-uremic causes such as hyperparathyroidism, malignancies or autoimmune diseases [70,71]. Its histopathological appearance is characterized by media calcification, endovascular fibrosis, intima hyperplasia and vascular thrombosis [72,73]. Additionally, ectopic calcium deposits can also be found in extravascular connective and adipose tissue [74] (see Table 5, Figure 4).

### 3.5. Diabetic Leg Ulcers

Diabetic patients combine multiple risk factors for the occurrence of lower extremity ulcers; diabetes itself is a risk factor for PAD, diabetic microangiopathy and peripheral neuropathy [1]. The involvement of diabetic microangiopathy in leg ulcers is debated [75]. Even in non-ulcerated skin, it has long been discussed whether histopathological changes in diabetes are secondary to PAD or primary to diabetic microangiopathy [76,77,78]. Histopathological patterns that can be associated specifically with diabetic microangiopathy are rare. Capillary thickening in non-ulcerated skin could hint to microangiopathic involvement in wound development, but is not only found in diabetic patients [76]. Fibrin cuffs, as seen in VLUs, have been both found in ischemic and non-ischemic diabetic ulcers [78].

## 4. Neuropathic Leg Ulcer

Neuropathic ulcers predominantly occur in diabetic patients as a result of peripheral neuropathy leading to insensitivity to pressure or trauma. Ulcerations frequently develop at common pressure points, such as the plantar surface of the foot [79]. Hyperkeratosis in marginal areas is an indispensable characteristic and tends to exceed the size of the underlying epidermal tissue [1,80]. Furthermore, the lesions present with a diffuse and intense inflammatory reaction represented by nodular leukocyte conglomerates. Cellular debris, a degraded extracellular matrix and necrosis can be observed, showing progressive dehydration toward the surface of the ulcer. The dermis is hypertrophic and fibrotic, thereby disrupting the normal structure of the extracellular matrix [80,81] (see Table 6). Impairment of larger blood vessels through wall thickening and capillary cuffs can be present, but it is neither specific nor mandatory [78].

## 5. Inflammatory Leg Ulcers

### 5.1. Vasculitis

Vasculitides are heterogeneous diseases that are characterized by primary inflammatory vessel damage, most commonly as a manifestation of a systemic autoimmune process. According to the Chapel Hill classification, they are classified based on the caliber of involved vessels as large-, medium- or small-vessel vasculitis [82]. In this context, ulcerations predominantly occur on the lower extremities and are most often caused by small vessel vasculitis [83]. In particular, anti-neutrophil cytoplasmic antibody (ANCA)-associated vasculitides—granulomatosis with polyangiitis, eosinophilic granulomatosis with polyangiitis, and microscopic polyangiitis—are prominent causes of vasculitic leg ulcers, albeit they are relatively rare diseases. However, several other forms, including secondary-type vasculitis, can also induce skin ulceration.

Granulomatosis with polyangiitis (formerly known as Wegener granulomatosis) frequently causes cutaneous lesions. Upon biopsy, there is fibrinoid necrosis of dermal small-vessel walls and nuclear debris. Perivascular inflammatory infiltrates consist of neutrophils, lymphocytes and eosinophils [84,85,86]. However, leukocytoclastic changes, necrotizing granulomatous and unspecific ulcerations, have also been reported [87,88]. A pyoderma gangrenosum-like appearance has also been reported [89,90].

Eosinophilic granulomatosis with polyangiitis (formerly Churg–Strauss Syndrome) often affects venules. Diagnosis is supported by numerous eosinophils that can be found in the inflammatory infiltrate, which can also consist of neutrophils, lymphocytes or macrophages [91,92].

In microscopic polyangiitis, tissues show infiltration of the vessel walls by neutrophils, as in leukocytoclastic vasculitis, reaching down into the deep dermis and subcutaneous fat tissue [93].

Cutaneous leukocytoclastic angiitis is one of few entities of single-organ vasculitis [82]. Fibrin deposits in the vessel wall, including strong perivascular neutrophilic infiltrates and cell debris, and extravasated erythrocytes are found regularly [94,95]. It can appear individually or as a cutaneous manifestation of other forms of vasculitis, such as ANCA-associated vasculitis, IgA vasculitis or cryoglobulinemic vasculitis [94]. Thus, the term leukocytoclastic vasculitis may describe the result of heterogeneous pathophysiological processes rather than a distinct entity [96].

Since secondary-type vasculitis can be the result of various heterogeneous diseases, such as infections or neoplasms, specific histopathologic patterns are absent. Small vessels are the most frequently affected, showing perivascular inflammation and fibrinoid necrosis of the vessel wall [97].

### 5.2. Pyoderma Gangrenosum

**Figure 5:**
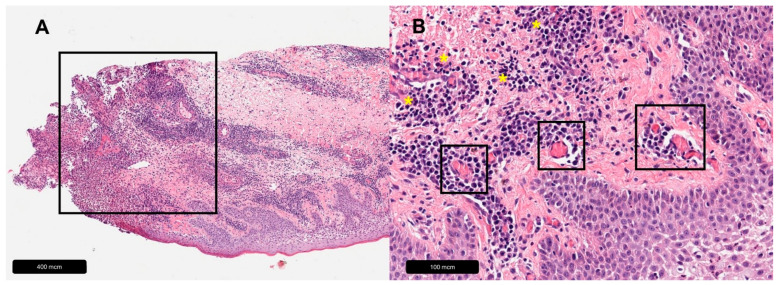
Pyoderma gangrenosum. (**A**) Overview, black rectangle = massive leukocyte infiltration. (**B**) Magnification, yellow asterisks = leukocyte infiltration, black rectangles = perivascular lymphocytic infiltrates with associated fibrin thrombi suggestive of secondary vasculitis.

Pyoderma gangrenosum (PD) is a very rare inflammatory disease that is characterized by deep necrotic ulcers [98]. The etiology is unknown; however, it is often associated with immune-mediated diseases, such as inflammatory bowel disease and rheumatoid arthritis [99]. The lower leg is the most commonly affected site. However, it can appear on the whole body; operation wounds are also predilected sites [100,101]. Diagnosis is challenging and often delayed, and misdiagnosis is frequent [52,102]. Histopathologic examination reveals dermal neutrophilia, perivascular lymphocytic infiltrate, endothelial swelling and necrosis [103,104,105]. However, histologic findings are non-specific and variable and depend on the clinical stage and the location of the lesion [18,106]. Previous reports have discussed the presence of vasculitis in PD ulcerations as secondary changes [18,107,108] (see Table 7, Figure 5).

### 5.3. Necrobiosis Lipoidica

Necrobiosis lipoidica is a necrotizing granulomatous inflammatory disease of the skin of unknown etiology, typically appearing on the anterior side of the lower leg [109,110]. The condition has been associated with diabetes mellitus [111]; however, recently, this association has been the subject of scientific research and discussion [112]. Histologically, a palisaded necrobiotic granuloma can be seen, consisting of degenerated bundles of collagen surrounded by histiocytes and multinucleate giant cells, creating an appearance that is sometimes described as “lasagna” or “cake layers” [111,112,113,114,115]. It involves the entire dermis and may extend into the subcutaneous fat resembling panniculitis [112,115]. The area is surrounded by a histiocytic and lymphocytic infiltrate. Focal loss of elastic tissue may also be seen [112,113] (see Table 8).

## 6. Decubitus

Decubitus ulcers result from soft tissue compression between a bony prominence and an external surface exceeding the capillary pressure for a prolonged period and are thus also referred to as pressure ulcerations. On the lower extremities, they are most frequently located on the heel and malleoli. Clinically, they are classified as grade I–IV [116]. In the general population, their prevalence is low; however, in long-term care settings such as geriatric nursing homes, the reported prevalence reaches 25–41% [2]. In past years, the prevalence has decreased due to pressure-reducing measures and increased mobilization [116]. Biopsies should be considered in nonhealing ulcers after 12 weeks of optimal care [15]. Histopathological analysis of the edges and marginal areas of clinically advanced decubitus ulcers (clinical grade IV) shows heterogenous features that can be summed up in four groups. Type I shows ulcer edema, occluded large blood vessels, degenerated fibroblasts and extensive neutrophils and macrophages. In type II, the surface consists of a fibrin coating and infiltrating neutrophils and macrophages, and the edge contains a considerable number of inflammatory cells. Blood vessels remain open. Type III is characterized by dense fibrinous regions with infiltrating inflammatory cells and remnants of vacuolated fibroblasts in the ulcer edge, and type IV demonstrates a dermal-type structure above a layer of subcutaneous fat. The first shows fat droplets and vacuolated fibroblasts, whereas the latter consists of few fibroblasts and inflammatory cells [117]. The ulcer center changes its appearance depending on the stage of disease. Early wounds exhibit a hemorrhagic crust, a perivascular lymphocytic infiltrate and a diffuse polymorphonuclear cell infiltrate. Healing decubitus ulcers show granulation tissue and edema as well as fibroblast and capillary proliferation. Long-standing, persistent decubitus ulcers are characterized by diffuse fibrosis, coagulation necrosis on the surface and the loss of epidermal appendages [118] (see Table 9).

## 7. Hydroxyurea-Induced Ulcers

Hydroxyurea is a cytotoxic agent that is commonly used to treat chronic myeloproliferative disorders and acute myeloid leukemia, but it also finds application in other neoplastic diseases as well as in nonproliferative disorders [119]. Hydroxyurea-induced ulcerations are predominantly found in the malleolar region and can often not be distinguished from VLUs macroscopically [120,121]. Histology shows pseudoepitheliomatous hyperplasia, epidermal spongiosis or atrophy in the margin area [119,122,123,124]. Dermal vessels exhibit endothelial cell swelling, thrombotic occlusions and thickening of the vessel walls. Perivascular lymphocytic inflammation with and without leukocytoclastic vasculitis has been reported [119,122,124,125,126]. In later stages, focal hyalinization of blood vessel walls, intraluminal fibrin deposition and dermal fibrosis can be seen, which may resemble the pathologies found in livedoid vasculopathy [119,122,123] (see Table 10).

## 8. Ulcerating Skin Tumors

Ulcerations stemming from skin and soft tissue tumors, such as basal cell carcinoma (BCC), squamous cell carcinoma (SCC), malignant melanoma, various cutaneous lymphomas and types of sarcoma, can be the first sign of disease in affected patients [2]. Skin metastases of solid organ tumors can also lead to ulceration [18]. Therefore, when patients present with a long-standing ulceration, a malignant process must be excluded [10]. Histopathological findings ultimately depend on the underlying pathogenesis. Differential diagnosis can often be difficult, as BCC and SCC can both initially present as an ulcer or develop as a secondary lesion in ulcerated or traumatized skin [127,128,129,130,131,132,133,134,135]. Similar reports have also been published for different types of sarcoma and cutaneous lymphomas [136,137,138,139,140].

## 9. Other Causes

In addition to the diseases described above, leg ulcers can be caused by a plethora of causes. Several hematological and hemostatic disorders; metabolic, autoimmune and dermatologic diseases; genetic diseases; trauma; or adverse drug reactions can cause lower extremity ulcers [1,2]. For instance, Klinefelter’s syndrome (XXY karyotype) is associated with lower leg ulcerations; an increased level of plasminogen activator inhibitor-1 is supposedly involved in its pathogenesis [141,142,143]. At another end of the spectrum, microorganisms and infectious diseases that can lead to direct tissue necrosis include β-hemolytic Streptococcus pyogenes, Staphylococcus aureus, tuberculosis, osteomyelitis and leishmaniosis [1,2].

## 10. Discussion

Chronic leg ulcers are one of the most common medical problems, affecting up to 5% of the population >65 years of age and posing an increasingly significant public health issue, with treatment costs reaching up to an estimate of USD 96.8 billion in the USA for all chronic wounds and £400–600 million in the UK for VLUs alone [5,144,145]. Therefore, precise and early diagnosis is of high interest. The majority of leg ulcers are of vascular or diabetic origin; however, in developing countries, trauma and infections are more common [146,147].

The differentiation of etiology-specific characteristics and secondary processes in leg ulcers is a diagnostic challenge. Pericapillary fibrin cuffs are non-specific but most frequently seen in VLUs [35,78]. Intraluminal calcium deposits have been described in vascular ulcers irrespective of the underlying pathology in wounds lasting 2 years and longer [80]. Leukocytoclastic vasculitis has been discussed to be concomitant with inflammation, explaining its occurrence in other types of vasculitis, pyoderma gangrenosum or hydroxyurea-induced ulcers [94,96,103,126]. A recent study regarding vasculitis in the marginal area of ulcers of various etiologies detected vasculitis in over 50% of cases, questioning the diagnostic value of the histopathological finding in the diagnosis of ulcer etiology [148]. Some authors even suggested that biopsies obtained from the ulcer bed instead of the recommended edge might lead to a false positive vasculitis diagnosis [95].

Correct sample obtainment is critical for histopathological examination. The biopsy should be performed on the border of the ulcer and should include dermal and hypodermal tissue [95,149,150]. Poor site selection and technique otherwise may frequently lead to a delayed or false diagnosis [151].

Histopathological features discussed in this review aim at reflecting the broad consensus. Nevertheless, certain characteristics remain debated in the expert literature or have not been conclusively investigated. This includes the differential diagnosis between calciphylaxis and the arteriolosclerotic ulcer of Martorell, for example, where the presence of calcium deposits within the lobular fat has been historically used to distinguish both entities, whereas more recent works indicate that the diagnostic value of this finding might be limited [152]. Another example is the histopathological diagnosis of pyoderma gangrenosum, which some authors consider ill advised due to its non-specific inflammatory features [99].

Lower extremity ulcers constitute a major burden on public health. Most ulcers do not require histopathological analysis and can be sufficiently treated based on clinical findings. However, with uncommon or uncommonly appearing ulcers, histopathological findings are critical for the correct diagnosis and also frequently for subsequent treatment. The hallmarks listed in this review might serve as guidance for clinical practice.

## Figures and Tables

**Table 1:** Venous leg ulcer characteristics.

Venous Leg Ulcer	
Location	Malleoli, pretibial
Specifics	Late chronic venous disease
Epidermis	Spongiosis
	Hyperkeratosis
	Acanthosis
Dermis	Inflammatory cell infiltration
	Diffuse edema
	Hemosiderophages
	Dermal sclerosis
	Fibrosis
	Collagen bundle degeneration
Hypodermis	Lipodermatosclerosis
Vessels	Fibrin cuffs
	Reduced capillary density
	Dilated capillaries
	Erythrocyte extravasation
	Ectatic lymph vessels

**Table 2:** Livedoid vasculopathy characteristics.

Livedoid Vasculopathy	
Location	Bilateral
Specifics	White scars
Epidermis	Spongiosis
	Atrophy
Dermis	Secondary inflammatory changes
	Subpapillary plexus thrombosis
Vessels	Endothelial edema
	Wall thickening
	Fibrin thrombi
	Subintimal hyalinization

**Table 3:** Arterial-ischemic leg ulcer characteristics.

Arterial-Ischemic Leg Ulcer	
Location	Toes, plantar
Specifics	Demarcated lesions
Epidermis	Necrosis
	Epidermal thinning
Dermis	Sclerosis
	Necrosis
Vessels	Thrombosis

**Table 4:** Arteriolosclerotic ulcer of Martorell characteristics.

Arteriolosclerotic Ulcer of Martorell	
Location	Lateral, lower leg
Specifics	Disproportional pain
Epidermis	Necrosis
	Acanthosis
Dermis	Necrosis
Vessels	Media hypertrophy
	Stenosis
	Calcification
	Sub-endothelial hyalinosis
	Thrombosis
	Subintimal hyalinization

**Table 5:** Calciphylaxis characteristics.

Calciphylaxis	
Location	Lower leg
Specifics	Disproportional pain
Epidermis	Necrosis
Dermis	Necrosis
	Calcium deposits
Hypodermis	Diffuse calcification
Vessels	Fibrosis
	Intima hyperplasia
	Media calcification
	Thrombosis

**Table 6:** Neuropathic leg ulcer characteristics.

Neuropathic Leg Ulcer	
Location	Plantar
Specifics	Diabetes-associated
Epidermis	Hyperkeratosis
	Epidermal thinning
Dermis	Leukocyte infiltration
	Degraded extracellular matrix
	Cellular debris
	Fibrosis
	Necrosis
Vessels	Wall thickening

**Table 7:** Pyoderma gangrenosum characteristics. PVLI = Perivascular lymphocytic infiltrate.

Pyoderma Gangrenosum	
Location	Lower leg
Specifics	Deep necrotic ulcers
Epidermis	Necrosis
Dermis	Neutrophil infiltration
Vessels	PVLI
	Endothelial swelling
	Secondary vasculitis

**Table 8:** Necrobiosis lipoidica characteristics.

Necrobiosis Lipoidica	
Location	Anterior, lower leg
Specifics	“Layered” histology
Epidermis	Necrosis
Dermis	Degenerated collagen
	Histiocytes
	Multinucleated giant cells
	Leukocyte infiltration

**Table 9:** Decubitus characteristics. PVLI = Perivascular lymphocytic infiltrate.

Decubitus	
Location	Heel, malleoli
Specifics	Histology type-dependent
Epidermis	-
Dermis	Fibrosis
	Leukocyte infiltration
	Atypical fibroblasts
	Edema
Vessels	PVLI
	Occlusion

**Table 10:** Hydroxyurea-induced ulcer characteristics. PVLI = Perivascular lymphocytic infiltrate.

Hydroxyurea-Induced Ulcers	
Location	Malleoli
Specifics	After hydroxyurea administration
Epidermis	Spongiosis
	Atrophy
	Hyperplasia
Dermis	Fibrosis
Vessels	Endothelial edema
	Wall thickening
	Thrombosis
	PVLI
	Secondary vasculitis

## Data Availability

The data used in this review can be shared upon reasonable request to the corresponding author.
